# Influence of Molecular Weight and Grafting Density of PEG on the Surface Properties of Polyurethanes and Their Effect on the Viability and Morphology of Fibroblasts and Osteoblasts

**DOI:** 10.3390/polym14224912

**Published:** 2022-11-14

**Authors:** Antonio David Abreu-Rejón, Wilberth Antonio Herrera-Kao, Alejandro May-Pat, Alejandro Ávila-Ortega, Nayeli Rodríguez-Fuentes, Jorge Alonso Uribe-Calderón, José Manuel Cervantes-Uc

**Affiliations:** 1 Centro de Investigación Científica de Yucatán, A.C, Unidad de Materiales, Calle 43 No. 130, Col. Chuburná de Hidalgo, Mérida C.P. 97205, Mexico; 2 Facultad de Ingeniería Química, Universidad Autónoma de Yucatán, Periférico Norte Km 33.5 Tablaje Catastral 13615, Chuburná de Hidalgo Inn, Mérida C.P. 97203, Mexico; 3 CONACYT-Centro de Investigación Científica de Yucatán, A.C, Unidad de Materiales, Calle 43 No. 130, Col. Chuburná de Hidalgo, Mérida C.P. 97205, Mexico

**Keywords:** PEG, viability, osteoblasts, fibroblasts, pH, polyurethane, polyethylene glycol

## Abstract

Grafting polyethylene glycol (PEG) onto a polymer’s surface is widely used to improve biocompatibility by reducing protein and cell adhesion. Although PEG is considered to be bioinert, its incorporation onto biomaterials has shown to improve cell viability depending on the amount and molecular weight (MW) used. This phenomenon was studied here by grafting PEG of three MW onto polyurethane (PU) substrata at three molar concentrations to assess their effect on PU surface properties and on the viability of osteoblasts and fibroblasts. PEG formed a covering on the substrata which increased the hydrophilicity and surface energy of PUs. Among the results, it was observed that osteoblast viability increased for all MW and grafting densities of PEG employed compared with unmodified PU. However, fibroblast viability only increased at certain combinations of MW and grafting densities of PEG, suggesting an optimal level of these parameters. PEG grafting also promoted a more spread cell morphology than that exhibited by unmodified PU; nevertheless, cells became apoptotic-like as PEG MW and grafting density were increased. These effects on cells could be due to PEG affecting culture medium pH, which became more alkaline at higher MW and concentrations of PEG. Results support the hypothesis that surface energy of PU substrates can be tuned by controlling the MW and grafting density of PEG, but these parameters should be optimized to promote cell viability without inducing apoptotic-like behavior.

## 1. Introduction

Grafting polyethylene glycol (PEG) onto polymeric materials has been widely used to improve their hydrophilicity and biocompatibility [1,2,3]. This is the case with polyurethanes (PUs), which are extensively used in biomedical applications due to their very good mechanical properties and safety [4]. However, the low hydrophilicity of most PUs reduces their biocompatibility, cell adhesion, and may lead to thrombosis [5,6,7,8]. Thus, PEG-grafted PU with improved hydrophilicity and biocompatibility have been reported [9,10,11,12,13]. The most accepted mechanism for increasing the PU biocompatibility through PEGylation is related to a volume exclusion effect created by water molecules bounded to grafted PEG on the substrate surface, which modulates the interaction of proteins and other molecules with a material surface [14,15,16]. The volume excluded depends mainly on the quantity of water molecules adsorbed by the PEG chains; thus, the main factors for regulating this exclusion effect are both PEG grafting density and the molecular weight (MW) of the PEG used [17]. Although a high volume exclusion can lead to a stealthing effect on the substrate, creating a bioinactive surface [18,19], the bioactivity of polymer surfaces can be maintained and even improved with an appropriate amount of PEG [20,21,22]. Therefore, by adjusting the MW and grafting density of PEG onto PU substrates, the cell response can be improved. For example, Wang et al. [23] and Tang et al. [24] reported that the viability of some types of cells increased with higher concentrations of PEG on PEGylated substrates. Additionally, Cai et al. [25] reported an increase in the adhesion, proliferation, and activity of MC3T3 osteoblasts onto PEG-grafted polypropylene fumarate, which increased with the grafting density of PEG up to a certain concentration; a further increase on the PEG grafting density decreased those parameters. On the other hand, Mao et al. [26] and Kasálková el al. [27] showed that higher MW of PEG on PEG-grafted copolymers promoted an increase in the viability of L929 fibroblasts and vascular smooth muscle cells, respectively.

Regardless of this, most works on PEGylated PUs have focused on the improvement of their biocompatibility by reducing the protein adsorption and cell adhesion [14,28,29,30,31,32]. However, the effect of the grafting density and MW of PEG on the surface properties and the increased viability exhibited by osteoblast and fibroblast cells in PEGylated PUs has not been reported. Thus, in this work, PEGylated PU substrates were synthesized to evaluate the effect of both PEG grafting density and MW on PU surface properties and on the viability and morphology of fibroblasts and osteoblasts.

## 2. Materials and Methods

### 2.1. Materials

Polyethylene glycol samples with molecular weights of 2 kDa, 6 kDa, and 10 kDa were supplied by Sigma-Aldrich Co. (Saint Louis, MO, USA); (referred as PEG2, PEG6, and PEG10, respectively). Hexamethylene diisocyanate (HMDI, 98%), triethylamine (TEA, 99%), toluene (99.5%), and tetrahydrofuran (THF, ≥99%) were also purchased from Sigma-Aldrich. Tecoflex™ SG-80A was obtained from Lubrizol (Wickliffe, OH, USA).

### 2.2. Polyurethane Substrates Preparation

PU substrates were prepared from segmented polyurethane Tecoflex™ SG-80A by solvent-casting technique. A solution of PU in THF (1:15 *w*/*v*) was magnetic stirred for 24 h at room temperature; this solution was then poured in Petri dishes and left for 48 h in an extraction hood. Then, the substrates were dried for 24 h at 60 °C in a vacuum oven.

### 2.3. PEG Grafting Modification of PU Substrates

**Figure 1:**
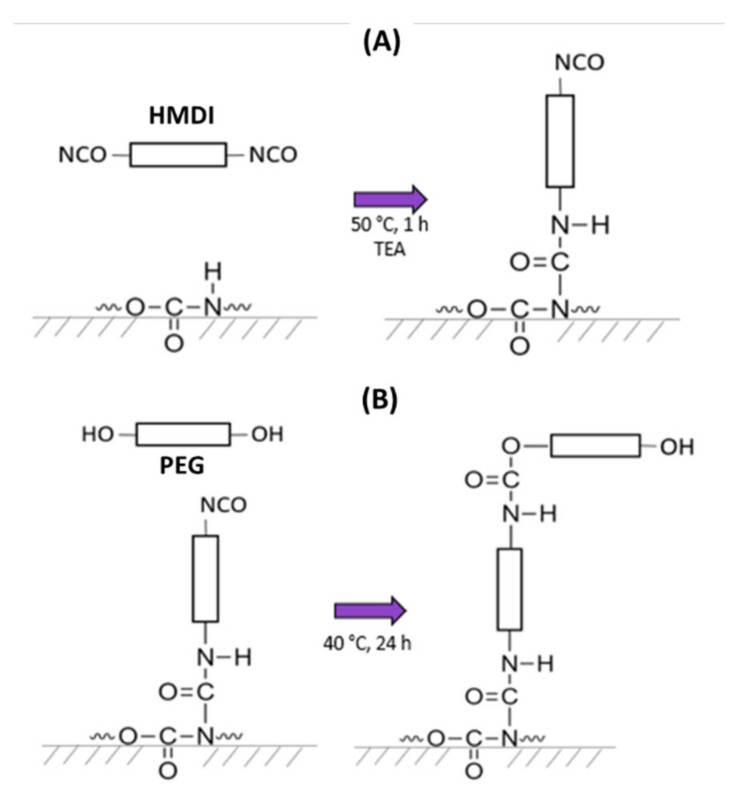
Grafting reaction of PEG onto PU substrates. (**A**): one isocyanate group of HMDI reacts with the secondary amine of the urethane group to yield allophanate linkages, with an isocyanate group remaining unreacted (first step); (**B**): one hydroxyl group from PEG reacts with the free isocyanate group obtained in the previous stage to yield a new urethane linkage (second step).

The grafting of PEG onto PU substrates was performed following the procedure reported by Freij-Larsson and Wesslén [33]; the reaction mechanism is illustrated in Figure 1. Briefly, a solution of HMDI/TEA (3:1 *v*/*v*) in toluene (30 mL) was heated to 50 °C; then, PU substrates were placed in the solution for 1 h with magnetic stirring under a nitrogen atmosphere. After this, substrates were rinsed with fresh toluene and immersed in a PEG-toluene solution (30 mL) at 40 °C for 24 h. Then, substrates were rinsed twice with fresh toluene to remove non-grafted PEG and left to dry in an extraction hood for 72 h. Finally, modified substrates were dried in a vacuum oven for 48 h at 55 °C to remove the residual toluene. HMDI and PEG (2 kDa, 6 kDa, or 10 kDa) were added at three different equimolar amounts (0.05, 0.10, and 0.15 mmol) per 1 g of PU.

### 2.4. Physicochemical Characterization

#### 2.4.1. Fourier Transform Infrared Spectroscopy (FTIR)

FTIR spectra of the substrates were obtained using a ThermoFisher Scientific Nicolet 8700 spectrometer equipped with an attenuated total reflectance (ATR) accessory of ZnSe (Madison, WI, USA). The analysis was performed from a 4000 cm^−1^ to 650 cm^−1^ spectral range with a resolution of 4 cm^−1^ averaging 100 scans.

#### 2.4.2. Thermogravimetric Analysis (TGA)

TGA curves were obtained using a Perkin-Elmer TGA 8000 equipment (Waltham, MA, USA). The heating rate used was 10 °C/min from 50 °C to 650 °C under a nitrogen atmosphere. The sample’s mass was 10 mg.

#### 2.4.3. Scanning Electron Microscopy (SEM)

The topography of the substrates was observed by scanning electron microscopy (SEM) with a JEOL JSM-6360LV (Akishima, Tokyo, Japan) microscope using an acceleration voltage of 20 kV. Samples were coated with a thin layer of gold and observed at 25 °C. Five regions from each sample were scanned.

#### 2.4.4. Contact Angle and Surface Free Energy Measurements

The static contact angles of sessile drops (5 µL) of distilled water, glycerol, and diiodomethane on PU substrata were measured in a ramé-hart 250-U1 goniometer (Succasunna, NJ, USA) at room temperature. The Van Oss and Good model was used to determine the surface free energy of the substrata, including dispersive and polar components, using the following equations [34,35]:

(1) 121+cosθγL=γSLWγLLW12+γS+γL−12+γS−γL+12

(2) γsAB=2γS+γS−12

where θ represents the contact angle, $\gamma_{L}$ is the surface free energy of liquid, $\gamma_{L}^{LW},\;\gamma_{L}^{-},\;\gamma_{L}^{+}$ are the corresponding dispersive, basic, and acid components of the surface free energy of the liquid, whereas $\gamma_{S}^{LW},\;\gamma_{S}^{AB},\;\gamma_{S}^{-},\;\gamma_{S}^{+}$ are the dispersive, polar, basic, and acid components of the solid surface free energy, respectively.

#### 2.4.5. Atomic Force Microscopy (AFM)

AFM images of the substrates’ surfaces were obtained with a Bruker INNOVA microscope (Santa Barbara, CA, USA) using a TESP nanoprobe silicone tip (spring constant of 42/Nm and 2 nm tip radius). The analysis was performed in tapping mode with a resonant frequency of 320 kHz and a scanning frequency of 0.3 Hz at room temperature. The scanning area (20 µm × 20 µm) was divided into four sub-areas (10 µm × 10 µm) from which roughness was obtained using the Nanoscope Analysis software. Five regions from each sample were scanned.

### 2.5. Cellular Studies

#### 2.5.1. Cell Culture

Mouse osteoblasts and human fibroblasts were maintained in Dulbecco’s Modified Eagle’s Medium (DMEM) supplemented with 10% fetal bovine serum, 1% penicillin-streptomycin at 37 °C, and 5% CO_2_; the osteoblastic medium contained 50 µg/mL of ascorbic acid. At 80% confluence, cells were detached with a solution of 0.25% trypsin-EDTA. Viable cells were counted using a mixture of trypan blue and cell suspension (1:1) in a hemocytometer (Neubauer cell chamber).

#### 2.5.2. Cell Viability

Viability of the cells was evaluated by indirect tests using extracts of the materials under standardized conditions (ISO 10993-5). PU substrates were UV-sterilized and washed with phosphate-buffered saline (PBS), and then placed in a culture medium at a 100:2.5 (mg/mL) ratio and incubated under standard culture conditions for 3 days. Cells were seeded in 96-well plates at a density of 5 × 10^3^ cells per well and incubated for 24 h in 100 µL of cell culture medium. After this, the culture medium was replaced with 100 µL of the extracts from the substrates and incubated for 24 h; cells cultured with regular culture medium were used as a control. Then, 20 µL of CellTiterBlue was added to the wells and incubated for 4 h. Afterward, absorbance was measured in a BioTek Cytation 3 plate reader (Winooski, VT, USA) at 570 nm and the viability of the cells was calculated with the following equation:

(3) $$Cell\;viability\;\left(\%\right)=\frac{A-A_{n}}{A_{p}-A_{n}}\times 100$$

where *A* is the absorbance of the test well and *A_p_* and *A_n_* are the absorbance of the positive and negative controls, respectively.

#### 2.5.3. Crystal Violet Staining

Cells from the viability assays were stained with crystal violet to observe their morphology; for this, the cells were rinsed twice with PBS and fixed with 50 µL of methanol. Then, the methanol was withdrawn and 50 µL of crystal violet solution (0.4%) was added to the wells and incubated for 10 min. Finally, the cells were rinsed with distilled water and observed in a Labomed TCM 400 microscope (Los Angeles, CA, USA).

### 2.6. Statistical Analysis

Data were analyzed by one-way ANOVA, with Tukey’s multiple comparison tests in the software Origin (2008) with a significance of *p* < 0.05.

## 3. Results

### 3.1. Spectroscopic Analysis

**Figure 2:**
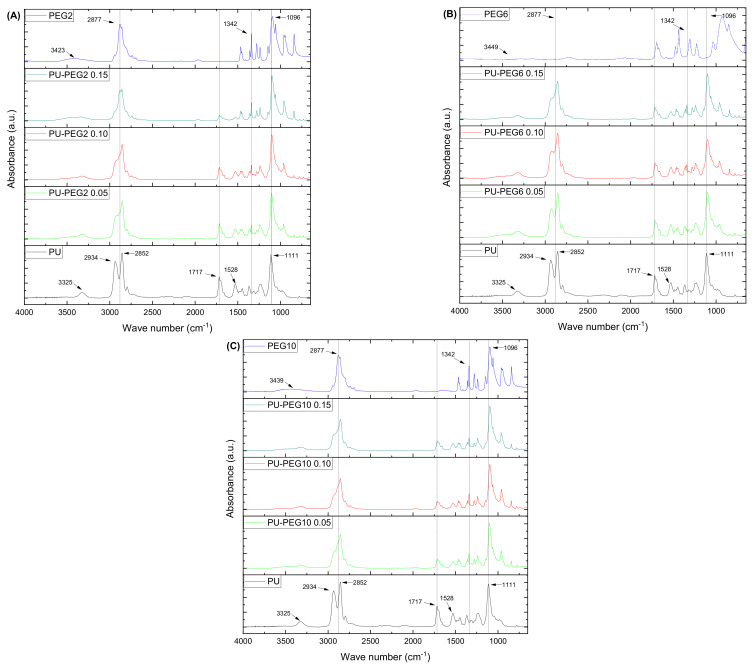
FTIR-ATR spectra of the PU substrata and PEG. As the grafting density is increased, the spectra of the substrata look more similar to PEG spectra, indicating that there is a higher amount of PEG chains present. (**A**): PEG 2 kDa; (**B**) PEG 6 kDa; (**C**) PEG 10 kDa.

FTIR spectra of the samples (untreated and treated polyurethane substrata, and PEG) are presented in Figure 2. Tecoflex™ exhibited bands at 3325 cm^−1^ (N–H stretching vibration), 2934 and 2852 cm^−1^ (CH_2_ asymmetric and symmetric stretching vibrations), 1717 cm^−1^ (C=O stretching vibration, amide I), 1528 cm^−1^ (amide II, C–N stretching bend and N–H in-plane deformation bend), and 1111 cm^−1^ (C–O asymmetric stretching vibration). On the other hand, the PEG spectra (PEG2, PEG6, and PEG10) showed the characteristic bands at 3423 cm^−1^ (O–H stretching vibration), 2877 cm^−1^ (C–H stretching vibration), 1342 cm^−1^ (O–H bending vibration), and 1096 cm^−1^ (C–O–C stretching vibration); no significant differences were detected among them. 

Interestingly, spectra of the modified PU substrata resemble that of PEG, as the PEG molar concentration is increased. In this manner, the grafted substrata show a decrease in the absorption bands located at 2934 and 1717 cm^−1^ and the appearance of a peak at 1342 cm^−1^ which is absent in the pristine PU. Additionally, the band at 1111 cm^−1^, related to the C–O stretching vibration, is shifted to lower wave numbers and the band at 2852 cm^−1^ shows the opposite behavior as the PEG concentration increases in the grafted substrata, which suggests that the amount of PEG on the modified substrata is increased. No bands are observed at the region of 2270–2250 cm^−1^ corresponding to the isocyanate group absorption, which indicates that there are no NCO residual groups from the grafting reaction.

A grafting reaction occurs on urethane bonds of PU, yielding allophanate bonds due to the reaction of an isocyanate from HMDI with the secondary amine of urethanes. Thus, in order to assess the formation of allophanate groups, a deconvolution analysis was performed on the carbonyl bands of the substrates (see Figure S1). As noted, the carbonyl band of the substrata is formed by the contribution of three absorption peaks located at 1719 cm^−1^, (A) associated with a free carbonyl stretching vibration of 1695 cm^−1^; (B) related to the hydrogen-bonded carbonyl stretching vibration, and 1663 cm^−1^; (C) due to the carbonyl from the urea-like substructure of allophanate groups [36,37]. The ratio C/(A + B) increased for all the grafted-PU compared to PU (see Table S1), which suggests that the number of allophanate linkages increased with respect to the urethane bonds.

### 3.2. Thermogravimetric Analysis

Degradation temperatures of PU, PEGs, and PEG-grafted substrata are reported in Table S2. PUs have a segmented structure: hard segments, formed by a diisocyanate and a chain extender, and soft segments that can be a polyol, polyether, or polyester [38]. Thus, PU is thermally degraded in two steps which were located at 340 and 413 °C; these stages are associated with the degradation of the hard segment (Td1) and the soft segment (Td2), respectively. On the other hand, PEGs are degraded in a single step at 405, 413, and 417 °C for PEG2, PEG6, and PEG10, respectively. In general, the thermal stability of the PEG-grafted substrata decreased with respect to PU; however, this effect is less appreciated as the MW and grafting density of PEG increased.

### 3.3. Surface Topography

**Figure 3:**
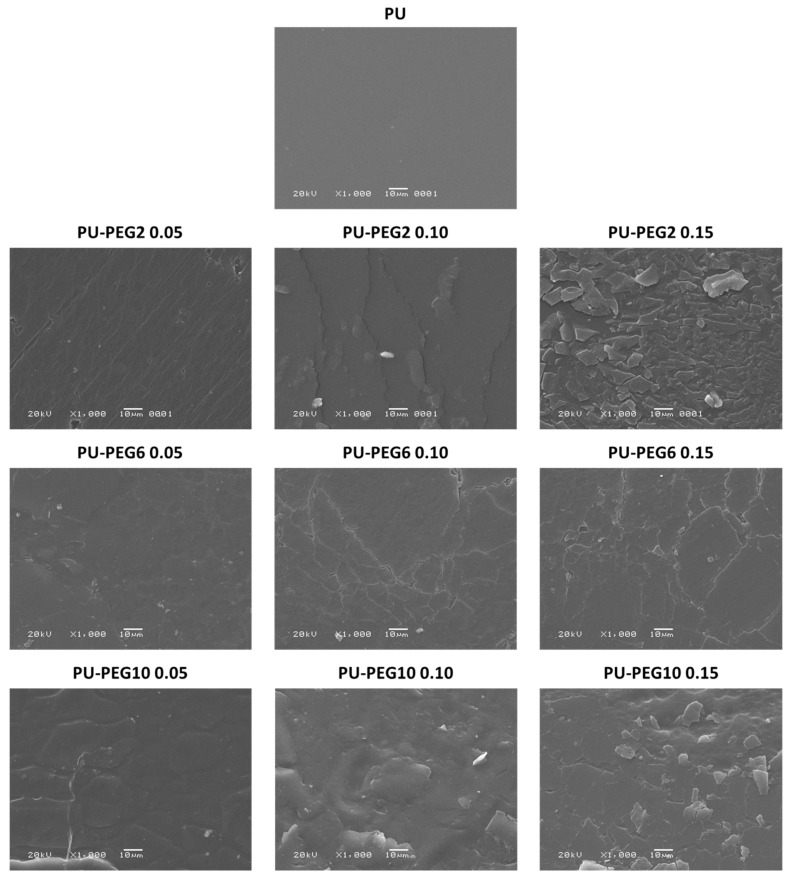
SEM images of the PU substrata. A PEG covering is formed on the surface of the substrates.

**Figure 4:**
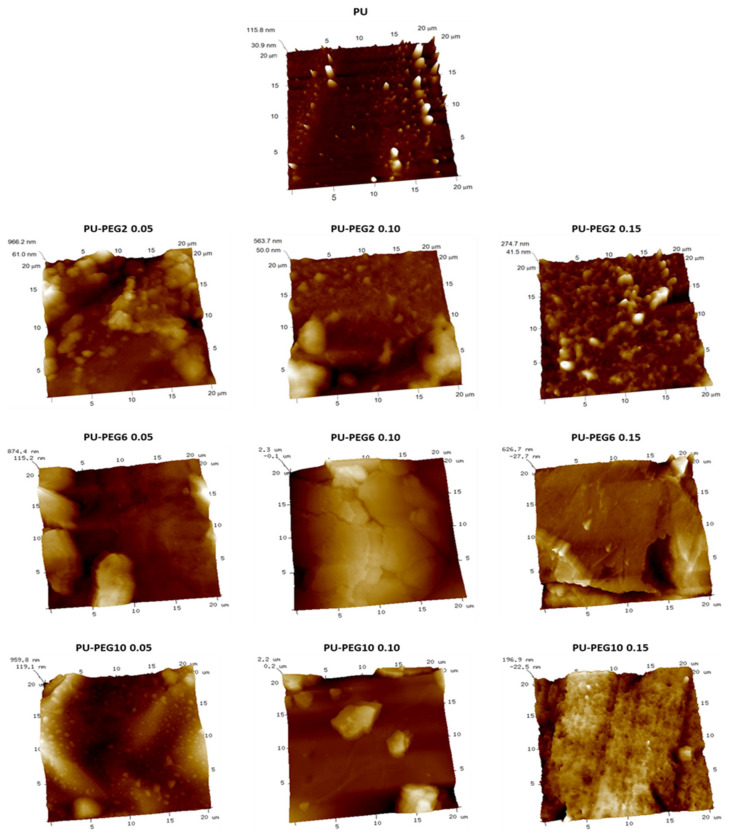
AFM images of the unmodified and grafted PU substrata.

SEM images (Figure 3) show the surface topology of all samples; unmodified PU revealed a smooth surface but PEG-modified PUs show rough surfaces depending on the MW and grafting density of PEG. In general, the topography of the substrata changed gradually from a slightly rough one at the lower PEG concentration (0.05 mmol) to topographies exhibiting cracks and ridges as the PEG grafting density increased, which suggests that the influence of the grafting density on the topography is higher than that of the MW of PEG. The AFM images in Figure 4 corroborate that the topology of the substrata becomes more irregular by the grafting of PEG. These observations are consistent for all regions observed by both SEM and AFM. It was also noted that roughness was increased in grafted surfaces in comparison to untreated PU substrate, but no clear effect of either concentration or Mw was observed; values obtained for non-modified PU, and PU-PEG2, PU-PEG6, and PU-PEG10 samples were 30 + 20 nm; 120 + 60 nm; 208 + 50 nm; 150 + 50 nm, respectively. 

### 3.4. Contact Angles and Surface Free Energy

**Figure 5:**
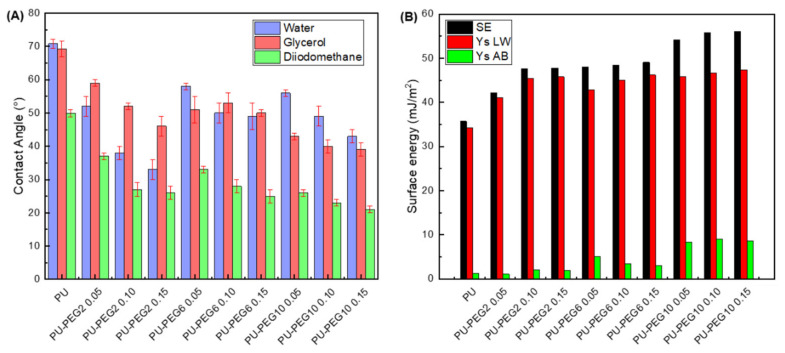
(**A**): Contact angles of the unmodified and PEG-grafted PU substrata with water, glycerol, and diiodomethane; (**B**): surface free energy of the substrata obtained with van Oss and Good model. SE: surface energy; Ys LW: dispersive component; Ys AB: polar component.

Contact angles of sessile drops on the substrata surfaces are presented in Figure 5A. Water and glycerol contact angles on PU are similar (~70°); however, diiodomethane exhibits lower values due to its non-polar characteristics. As PEG was grafted onto the PU surface, the values of the contact angle for all testing liquids diminished; even more, the values tend to decrease with grafting density for all MW of PEG. The decrease in the contact angles of sessile drops of water and glycerol suggests that the hydrophilicity of the substrates increased with the PEG grafting concentration. This characteristic was more evident when the molecular weight of PEG was lower (PEG2), probably due to the fact that PEG molecules are more soluble as the MW is lower. It should be mentioned that PEG required a longer time to dissolve both in water and toluene as MW increased. On the other hand, the decrease in contact angles with diiodomethane sessile drops suggest that the hydrophobic affinity of substrata was also increased. Figure 5B shows the calculated total surface free energy of the substrata, which increased with the grafting density and MW of PEG. Moreover, polar and dispersive components were also increased with the concentration and MW of PEG.

### 3.5. Cell Viability and Morphology

**Figure 6:**
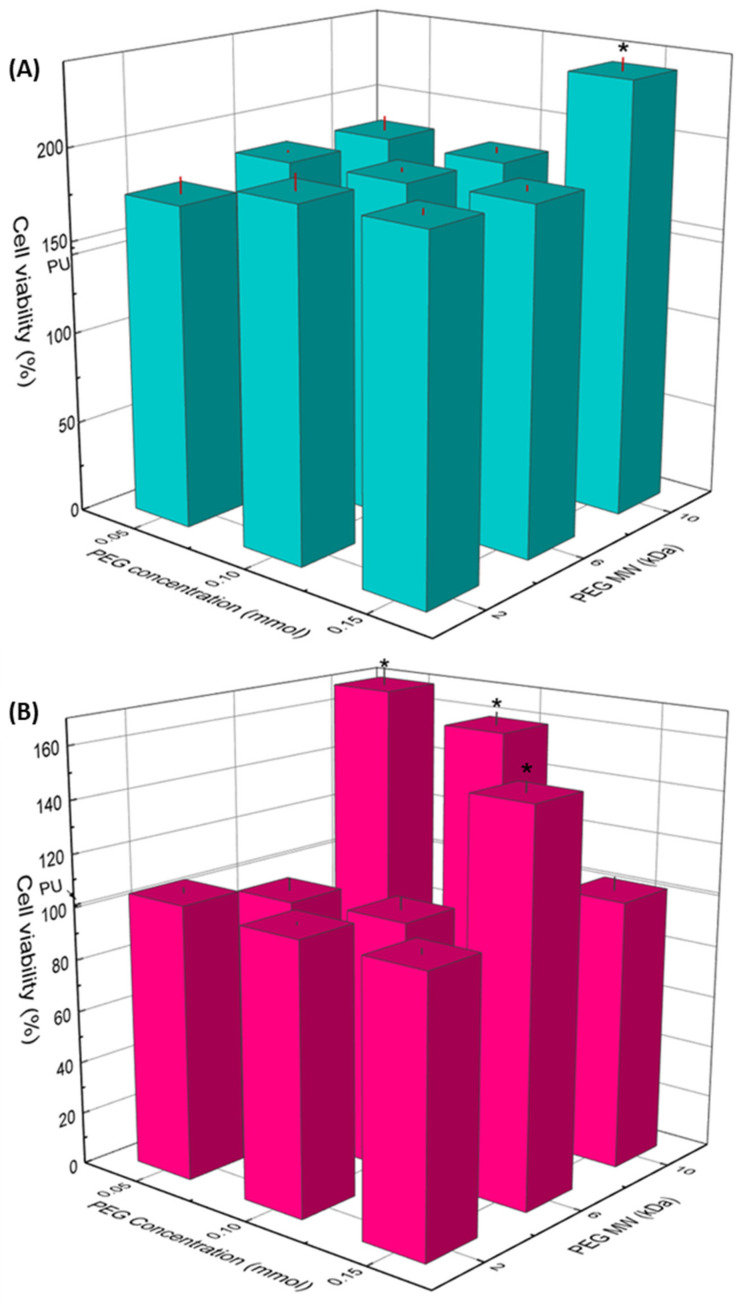
Cell viability of osteoblast (**A**) and fibroblast (**B**) cells by indirect contact with PU and PEG-grafted substrata for 24 h. (*): Statistical difference (*p* < 0.05).

The viability of osteoblasts and fibroblasts in contact with extracts of the substrata is presented in Figure 6A,B, respectively. As it can be seen, none of the samples exhibited a lower viability than the cell culture without exposition to the extracts (CWE), which was taken as reference of 100% viability. It is interesting to note that osteoblasts presented higher viability than that of CWE for all the substrates; even more, grafted substrata yielded higher viability than unmodified PU. However, no significant difference between the distinct concentrations and MW of PEG was observed on osteoblast viability; only PU-PEG10 0.15 promoted a higher viability compared with the other PEG-grafted substrata. On the other hand, the viability of fibroblasts was not affected, neither by the lower MW (2 kDa) nor the lower concentrations (0.05 and 0.10 mmol) of intermediate MW (6 kDa) of PEG; nevertheless, it increased for PU-PEG6 0.15. The highest MW of PEG (10 kDa) promoted a higher fibroblasts viability, but it decreased as the grafting density increased.

**Figure 7:**
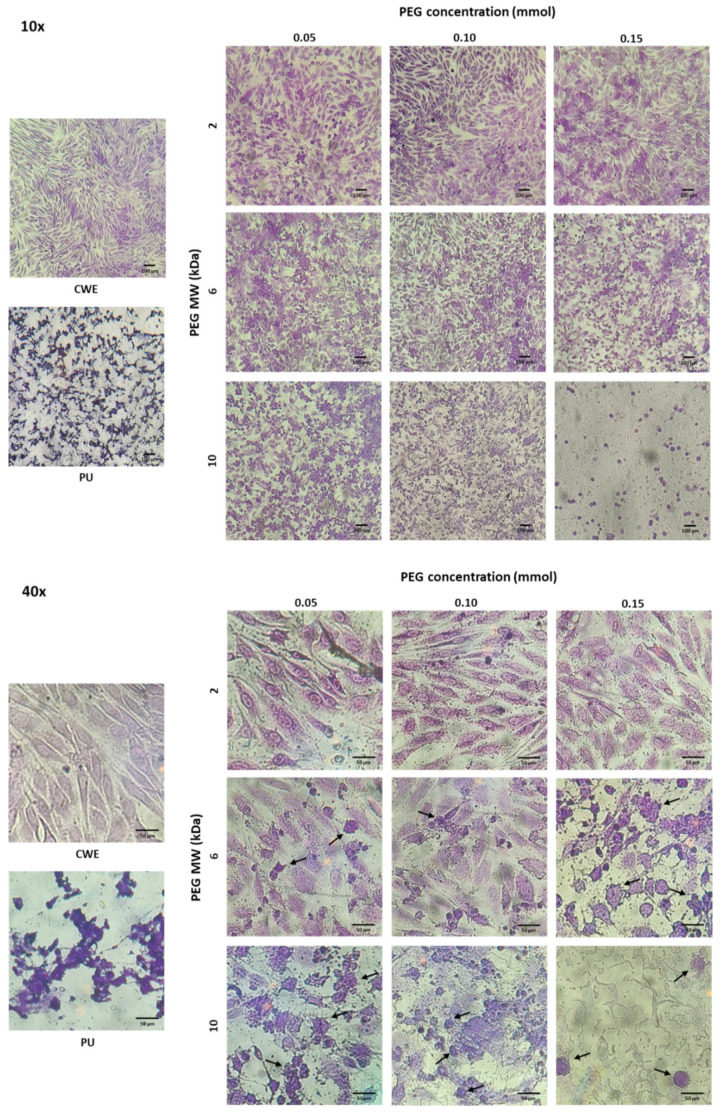
Microscopic images of crystal violet-stained osteoblasts in contact with extracts of the PEG-grafted PU substrata. Increasing MW and grafting densities of PEG promotes a higher number of rounded cells. CWE: cells without exposition to the extracts. Arrows indicate cells with membrane blebbing.

**Figure 8:**
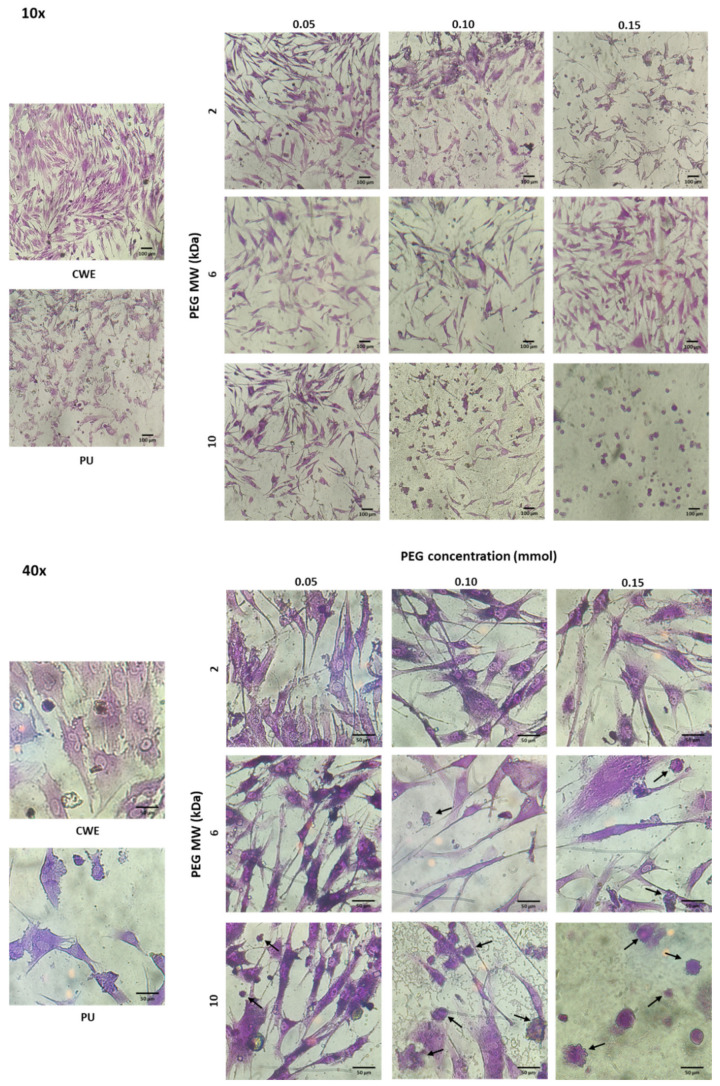
Microscopic images of crystal violet-stained fibroblasts in contact with extracts of the PEG-grafted PU substrata. Increasing MW and grafting densities of PEG promotes a higher number of rounded cells. CWE: cells without exposition to the extracts. Arrows indicate cells with membrane blebbing.

Although none of the extracts were cytotoxic, the morphology of osteoblasts and fibroblasts was different when cells were cultured with extracts of these grafted substrata (see Figure 7 and Figure 8). In this sense, osteoblasts and fibroblasts presented an irregular and shrunken morphology when these cells were brought into contact with the extracts derived from unmodified PU. On the other hand, when these cells were cultured with the extracts of the PEG-grafted PUs, they exhibited a more extended morphology, similar to that of CWE; however, some cells showed a rounded morphology with PEG-grafted substrata. Interestingly, the number of rounded cells increased with the increment in the MW and grafting density of PEG; even more, the number of cells was reduced with PU-PEG10 0.15 extracts and all these cells presented a round shape. It should be mentioned that some of the round-shaped cells also presented membrane blebbing as shown in Figure 7—40× and Figure 8—40×, which seems to indicate that these cells were in apoptosis. Interestingly, more rounded and apoptotic-like cells can be observed as MW and grafting density of PEG increase, while the number of viable cells is reduced.

## 4. Discussion

As mentioned before, the two main factors that influence the surface properties of PEGylated surfaces are grafting density and MW of PEG; thus, the effect that these parameters have on the surface properties of PU substrata as well as on the viability and morphology of osteoblasts and fibroblasts was investigated. For this, a two-step reaction was used to graft PEGs of 2, 6, and 10 kDa onto PU substrates at the molar concentrations of 0.05, 0.10, and 0.15 mmol/g. We previously demonstrated that the grafting reaction took place and PEG was successfully grafted onto PU substrates by the modification of the urethane linkages with an isocyanate group of HMDI, yielding allophanate groups [39]. 

FTIR spectra revealed the incorporation of PEG chains onto the PU substrata. The spectra of the grafted substrates were more similar to that of PEG as the molar concentration of PEG increased, which confirms that the grafting density increased. The formation of allophanate linkages in the grafted PUs was also confirmed by the increase of the intensity of the IR band associated to these groups, and by the reduction on their thermal stability with respect to unmodified PU [40]. The decrease in Td1 of the grafted substrata was lower as the MW of PEG increased due to the higher thermal energy required to decompose larger molecules. This suggests that the amount of PEG on the substrates was increased. 

As expected, the hydrophilicity and surface free energy of PEG-grafted substrata were higher than for unmodified PU, due to the PEG covering on their surfaces being revealed by SEM and AFM analysis. The increment in the hydrophilicity of the substrata would be due to the increasing amount of PEG on the surface of PU substrata that introduces more ether groups; in addition, the formation of more urethane and allophanate groups in the grafting reaction could contribute to the hydrophilicity changes of the substrata. A PEG covering onto PU substrata became more irregular as the MW and grafting density of PEG increased. This effect is a consequence of the higher density and larger PEG chains that increase the mass of the covering on the substrates (confirmed by FTIR and TGA analysis) that creates bigger PEG domains on the surface of PU substrata as observed by AFM. Although rugosity of a surface can affect contact angle measurements, in this case, the rugosity is due to the PEG on PU substrata which is soluble in the three solvents used, so the rugosity effect could be neglected. The increment in both polar and dispersive components of the surface free energy are due to the amphiphilic nature of PEG, which contains polar (ether group that acts as a basic polar domain) and non-polar (–CH_2_–CH_2_–) domains [41,42]. Based on these results, it is clear that the surface characteristics of PU can be tailored by choosing the MW and the grafting density of anchored PEG.

Results obtained from the biological characterizations revealed a positive effect of PEG on the viability of osteoblasts and fibroblasts. These results also show that the MW and concentration of PEG seems to be affecting the viability of the cells. In a previous work, we found that the increase in the viability of osteoblasts was caused by an increase in the pH of culture medium related to PEG concentration, and this effect was due to the fact that PEG interacts with CO_2_ molecules [39]. Therefore, an experiment was conducted to evaluate the effect of PEG MW on the pH of the culture media. Thus, PEG of the three MW used for the preparation of the grafted substrata were added to DMEM at three mass concentrations (1, 2, and 3 mg/mL) and the pH of the culture mediums was measured; data are presented in Table S3. It was found that as the PEG MW increased, lower molar concentrations led to similar pH increments than those exerted by PEG of the lower MW (PEG2). This could be related to the number of ether groups present in the medium, i.e., as the MW of PEG increases, the number of ether groups contained in a single PEG molecule will increase due to the higher number of repetitive units (–CH_2_–CH_2_–O–). Hence, smaller molar concentrations would cause a similar number of PEG-CO_2_ interactions in the cell culture medium. In consequence, PEG chains released by the grafted substrata would have a different impact on the pH of the culture medium depending on the MW of PEG, yielding a more alkaline pH for a higher MW. As bones function as an alkaline buffer reserve, osteoblasts participate in the acid-base homeostasis [43]; therefore, osteoblasts become more active at an alkaline microenvironment, which results in a higher viability. On the other hand, fibroblasts do not participate in pH homeostasis in the way osteoblasts do; however, they are still affected by pH variations [44,45], but the underlying mechanism by which it happens is different. It has been reported that human fibroblasts can tolerate and proliferate in an alkaline pH, and that the optimal pH for their growth ranges from 7.5 to 7.8 [46,47]. This would explain why the viability of fibroblasts increased with PU-PEG6 0.15, PU-PEG10 0.05, and PU-PEG10 0.10 samples, as the pH of the extracts from these samples might fall in the optimal range for fibroblast growth.

In spite of the viability results, the morphology of the cells was affected, suggesting the presence of apoptotic events in some cells by the increment in MW and grafting density of PEG. This could be due to the pH increment discussed before, as it would put the cells in a state that is out of physiological conditions, which stresses them. Similar to what we found, Lie et al. [48] reported a higher growth of human fibroblasts at a pH around 7.8, but also that their morphology was affected by an increased pH, showing the accumulation of membrane-bound bodies and autophagic vacuoles, characteristic of apoptotic processes. One possible reason for these effects (increased viability but apoptotic morphology) besides the fact that some enzymes and growth factors have an optimal activity at an alkaline pH, is related to the influence of pH on intracellular Ca^2+^ concentration ([Ca^2+^]_i_). Alkaline pH values have shown to raise [Ca^2+^]_i_ levels due to an upregulated activity of Ca^2+^ channels at the cell membrane [49,50,51]. This higher [Ca^2+^]_i_ can increase mitochondrial activity, leading to higher viability and proliferation of the cells; however, increased levels of Ca^2+^ that are sustained for long periods or Ca^2+^ overloads can also trigger apoptotic mechanisms due to an increase in the generation of reactive oxygen species (ROS) and the activation of caspases [49,52,53,54]. Therefore, though an alkaline pH can induce apoptosis as the results suggest, the viability of the cells would not be decreased since it stimulates growth and activity of osteoblasts and fibroblasts. Nonetheless, more studies are required to corroborate this hypothesis.

Overall, results show that both the grafting density and MW of grafted-PEG have an influence on the different surface properties of PU substrata and on the viability and morphology of cells. However, it is interesting to mention a different approach for surface modification that involves the grafting of short-branched molecules [55,56,57]. These molecules that have a non-linear structure create more complex interactions with the surrounding media; thus, the effect on surface properties, as well as their biological effects on cells, could be different. Then, it would be interesting to further investigate the effects of grafting more complex molecules onto PU and other polymer surfaces.

## 5. Conclusions

PU substrates were successfully modified by the grafting of PEG of different MW and at different molar concentrations. FTIR spectroscopy confirmed the presence of PEG on the modified substrates and that the grafting reaction occurred at the urethane bonds of the hard segment of PU; this was also confirmed by TGA results. It was also found that PEG grafting generated a covering onto PU substrata which modified their topography and increased their hydrophilicity and surface free energy as the MW and concentration of PEG increased. 

PEG grafting improved the biocompatibility of PU, which is supported mainly by the higher viability exhibited by osteoblasts as well as by the healthier morphology presented by both fibroblasts and osteoblasts cultured using extracts of the materials. Results also show that the pH of a culture medium increases due to both MW and grafting density of PEG, which was the reason for the increased cell viability; however, this could potentially trigger apoptosis on cells. Finally, it was demonstrated that the surface free energy, the topography, and cell behavior of PU substrata can be tuned by the MW and density of PEG grafted onto them.

## Data Availability

Not applicable.

## Supplementary Materials

The following supporting information can be downloaded at: https://www.mdpi.com/article/10.3390/polym14224912/s1, Figure S1: Deconvolution of the carbonyl bands from unmodified and PEG-grafted substrata; Table S1: Band ratios of carbonyl bands from unmodified and PEG-grafted substrata; Table S2: Decomposition temperatures (Td) of PU, PEG, and PEG-grafted substrata; Table S3: pH measurements of culture medium (DMEM) with the PEG diluted.

## References

[1] Cheng C.C., Mi F.L., Hsu S.H., Don T.M. (2014). Structure Characterizations and Protein Resistance of Chitosan Membranes Selectively Crosslinked by Poly(Ethylene Glycol) Dimethacrylate. Cellulose.

[2] Ren Z., Chen G., Wei Z., Sang L., Qi M. (2013). Hemocompatibility Evaluation of Polyurethane Film with Surface-Grafted Poly(Ethylene Glycol) and Carboxymethyl-Chitosan. J. Appl. Polym. Sci..

[3] Bozuyuk U., Dogan N.O., Kizilel S. (2018). Deep Insight into PEGylation of Bioadhesive Chitosan Nanoparticles: Sensitivity Study for the Key Parameters Through Artificial Neural Network Model. ACS Appl. Mater. Interfaces.

[4] Wang W., Wang C., Davim J.P. (2012). Polyurethane for Biomedical Applications: A Review of Recent Developments. The Design and Manufacture of Medical Devices.

[5] Jung J.-H., Choi C.-H., Chung S., Chung Y.-M., Lee C.-S. (2009). Microfluidic Synthesis of a Cell Adhesive Janus Polyurethane Microfiber. Lab Chip.

[6] Lehle K., Stock M., Schmid T., Schopka S., Straub R.H., Schmid C. (2008). Cell-Type Specific Evaluation of Biocompatibility of Commercially Available Polyurethanes. J. Biomed. Mater. Res. Part B Appl. Biomater..

[7] Zhou X., Zhang T., Guo D., Gu N. (2014). A Facile Preparation of Poly(Ethylene Oxide)-Modified Medical Polyurethane to Improve Hemocompatibility. Colloids Surf. A Physicochem. Eng. Asp..

[8] Zhou X., Zhang T., Jiang X., Gu N. (2009). The Surface Modification of Medical Polyurethane to Improve the Hydrophilicity and Lubricity: The Effect of Pretreatment. J. Appl. Polym. Sci..

[9] Noorisafa F., Razmjou A., Emami N., Low Z., Korayem H., Kajani A.A. (2016). Surface Modification of Polyurethane via Creating a Biocompatible Superhydrophilic Nanostructured Layer: Role of Surface Chemistry and Structure. J. Exp. Nanosci..

[10] Shih T.-Y., Yang J.-D., Chen Y.-H., Hong C.-W., Yang M.-J., Chen J.-H. (2013). Development of Peg-Containing Brush Copolymer: Their Effect on Resistance to Protein Adsorption Behaviors. Biomed. Eng. Appl. Basis Commun..

[11] Røn T., Javakhishvili I., Jeong S., Jankova K., Lee S. (2021). Low Friction Thermoplastic Polyurethane Coatings Imparted by Surface Segregation of Amphiphilic Block Copolymers. Colloid Interface Sci. Commun..

[12] Chen K., Zhou S., Wu L. (2015). Self-Repairing Nonfouling Polyurethane Coatings via 3D-Grafting of PEG-b-PHEMA-b-PMPC Copolymer. RSC Adv..

[13] Jung I.K., Bae J.W., Choi W.S., Choi J.H., Park K.D. (2009). Surface Graft Polymerization of Poly(Ethylene Glycol) Methacrylate onto Polyurethane via Thiol–Ene Reaction: Preparation and Characterizations. J. Biomater. Sci. Polym. Ed..

[14] Lee H., Lee K.D., Pyo K.B., Park S.Y., Lee H. (2010). Catechol-Grafted Poly(Ethylene Glycol) for PEGylation on Versatile Substrates. Langmuir.

[15] Wattendorf U., Merkle H.P. (2008). PEGylation as a Tool for the Biomedical Engineering of Surface Modified Microparticles. J. Pharm. Sci..

[16] Jiang Z., Feng X., Zou H., Xu W., Zhuang X. (2021). Poly(l-Glutamic Acid)-Cisplatin Nanoformulations with Detachable PEGylation for Prolonged Circulation Half-Life and Enhanced Cell Internalization. Bioact. Mater..

[17] Wattendorf U., Koch M.C., Walter E., Vörös J., Textor M., Merkle H.P. (2006). Phagocytosis of Poly(L-Lysine)-Graft-Poly (Ethylene Glycol) Coated Microspheres by Antigen Presenting Cells: Impact of Grafting Ratio and Poly (Ethylene Glycol) Chain Length on Cellular Recognition. Biointerphases.

[18] Xu X., Tang J., Han Y., Wang W., Chen H., Lin Q. (2016). Surface PEGylation of Intraocular Lens for PCO Prevention: An in Vivo Evaluation. J. Biomater. Appl..

[19] Tsai W., Chen Y., Chien H. (2009). Collaborative Cell-Resistant Properties of Polyelectrolyte Multilayer Films and Surface PEGylation on Reducing Cell Adhesion to Cytophilic Surfaces. J. Biomater. Sci. Polym. Ed..

[20] Sun M., Deng J., Tang Z., Wu J., Li D., Chen H., Gao C. (2014). A Correlation Study of Protein Adsorption and Cell Behaviors on Substrates with Different Densities of PEG Chains. Colloids Surf. B Biointerfaces.

[21] Zhou G., Ma C., Zhang G. (2011). Synthesis of Polyurethane-g-Poly(Ethylene Glycol) Copolymers by Macroiniferter and Their Protein Resistance. Polym. Chem..

[22] Ma Y., Zhang W., Wang Z., Wang Z., Xie Q., Niu H., Guo H., Yuan Y., Liu C. (2016). PEGylated Poly(Glycerol Sebacate)-Modified Calcium Phosphate Scaffolds with Desirable Mechanical Behavior and Enhanced Osteogenic Capacity. Acta Biomater..

[23] Wang Y., Wu H., Wang Z., Zhang J., Zhu J., Ma Y., Yang Z., Yuan Y. (2019). Optimized Synthesis of Biodegradable Elastomer PEGylated Poly(Glycerol Sebacate) and Their Biomedical Application. Polymers.

[24] Tang G.P., Zeng J.M., Gao S.J., Ma Y.X., Shi L., Li Y., Too H.-P., Wang S. (2003). Polyethylene Glycol Modified Polyethylenimine for Improved CNS Gene Transfer: Effects of PEGylation Extent. Biomaterials.

[25] Cai L., Wang K., Wang S. (2010). Poly(Ethylene Glycol)-Grafted Poly(Propylene Fumarate) Networks and Parabolic Dependence of MC3T3 Cell Behavior on the Network Composition. Biomaterials.

[26] Mao S., Shuai X., Unger F., Wittmar M., Xie X., Kissel T. (2005). Synthesis, Characterization and Cytotoxicity of Poly(Ethylene Glycol)-Graft-Trimethyl Chitosan Block Copolymers. Biomaterials.

[27] Kasálková N., Makajová Z., Pařízek M., Slepička P., Kolářová K., Bačáková L., Hnatowicz V., Švorčík V. (2010). Cell Adhesion and Proliferation on Plasma-Treated and Poly(Ethylene Glycol)-Grafted Polyethylene. J. Adhes. Sci. Technol..

[28] Alibeik S., Zhu S., Yau J.W., Weitz J.I., Brash J.L. (2012). Modification of Polyurethane with Polyethylene Glycol–Corn Trypsin Inhibitor for Inhibition of Factor Xlla in Blood Contact. J. Biomater. Sci. Polym. Ed..

[29] Dennes T.J., Schwartz J. (2008). Controlling Cell Adhesion on Polyurethanes. Soft Matter.

[30] Xu L.-C., Siedlecki C.A. (2017). Protein Adsorption, Platelet Adhesion, and Bacterial Adhesion to Polyethylene-Glycol-Textured Polyurethane Biomaterial Surfaces. J. Biomed. Mater. Res. Part B Appl. Biomater..

[31] Ma N., Cao J., Li H., Zhang Y., Wang H., Meng J. (2019). Surface Grafting of Zwitterionic and PEGylated Cross-Linked Polymers toward PVDF Membranes with Ultralow Protein Adsorption. Polymer.

[32] Wendels S., Avérous L. (2021). Biobased Polyurethanes for Biomedical Applications. Bioact. Mater..

[33] Freij–Larsson C., Wesslén B. (1993). Grafting of Polyurethane Surfaces with Poly(Ethylene Glycol). J. Appl. Polym. Sci..

[34] McCafferty E., Wightman J.P. (1999). Determination of the Acid-Base Properties of Metal Oxide Films and of Polymers by Contact Angle Measurements. J. Adhes. Sci. Technol..

[35] Gudipati C.S., Finlay J.A., Callow J.A., Callow M.E., Wooley K.L. (2005). The Antifouling and Fouling-Release Perfomance of Hyperbranched Fluoropolymer (HBFP)-Poly(Ethylene Glycol) (PEG) Composite Coatings Evaluated by Adsorption of Biomacromolecules and the Green Fouling Alga *Ulva*. Langmuir.

[36] Mattia J., Painter P. (2007). A Comparison of Hydrogen Bonding and Order in a Polyurethane and Poly(Urethane−urea) and Their Blends with Poly(Ethylene Glycol). Macromolecules.

[37] Zimmerer C., Nagel J., Steiner G., Heinrich G. (2016). Nondestructive Molecular Characterization of Polycarbonate–Polyvinylamine Composites after Thermally Induced Aminolysis. Macromol. Mater. Eng..

[38] Guignot C., Betz N., Legendre B., Moel A.L., Yagoubi N. (2001). Degradation of Segmented Poly(Etherurethane) Tecoflex^®^ Induced by Electron Beam Irradiation: Characterization and Evaluation. Nucl. Instrum. Methods Phys. Res. Sect. B Beam Interact. Mater. Atoms.

[39] Abreu-Rejón A.D., Herrera-Kao W., May-Pat A., Ávila-Ortega A., Rodríguez-Fuentes N., Uribe-Calderón J.A., Cervantes-Uc J.M. (2022). Effect of PEG Grafting Density on Surface Properties of Polyurethane Substrata and the Viability of Osteoblast and Fibroblast Cells. J. Mater. Sci. Mater. Med..

[40] Shufen L., Zhi J., Kaijun Y., Shuqin Y., Chow W.K. (2006). Studies on the Thermal Behavior of Polyurethanes. Polym. Plast. Technol. Eng..

[41] Good R.J., van Oss C.J. (1992). The Modern Theory of Contact Angles and the Hydrogen Bond Components of Surface Energies. Mod. Approaches Wettability.

[42] Chao Y.C., Su S.K., Lin Y.W., Hsu W.T., Huang K.S. (2012). Interfacial Properties of Polyethylene Glycol/Vinyltriethoxysilane (PEG/VTES) Copolymers and Their Application to Stain Resistance. J. Surfactants Deterg..

[43] Galow A.-M., Rebl A., Koczan D., Bonk S.M., Baumann W., Gimsa J. (2017). Increased Osteoblast Viability at Alkaline PH in Vitro Provides a New Perspective on Bone Regeneration. Biochem. Biophys. Rep..

[44] Bumke M.A., Neri D., Elia G. (2003). Modulation of Gene Expression by Extracellular PH Variations in Human Fibroblasts: A Transcriptomic and Proteomic Study. Proteomics.

[45] Jones E.M., Cochrane C.A., Percival S.L. (2015). The Effect of PH on the Extracellular Matrix and Biofilms. Adv. Wound Care.

[46] Yin Y., Jian L., Li B., Liang C., Han X., Zhao X., Wang D. (2021). Mg-Fe Layered Double Hydroxides Modified Titanium Enhanced the Adhesion of Human Gingival Fibroblasts through Regulation of Local PH Level. Mater. Sci. Eng. C.

[47] Eagle H. (1973). The Effect of Environmental PH on the Growth of Normal and Malignant Cells. J. Cell. Physiol..

[48] Lie S.O., Schofield B.H., Taylor H.A., Doty S.B. (1973). Structure and Function of the Lysosomes of Human Fibroblasts in Culture: Dependence on Medium PH. Pediatr. Res..

[49] Schreiber R. (2005). Ca^2+^ Signaling, Intracellular PH and Cell Volume in Cell Proliferation. J. Membr. Biol..

[50] Nitschke R., Riedel A., Ricken S., Leipziger J., Benning N., Fischer K.-G., Greger R. (1996). The Effect of Intracellular PH on Cytosolic Ca^2+^ in HT_29_ Cells. Pflügers Arch. Eur. J. Physiol..

[51] De Souza C.N., Breda L.C.D., Khan M.A., de Almeida S.R., Câmara N.O.S., Sweezey N., Palaniyar N. (2018). Alkaline PH Promotes NADPH Oxidase-Independent Neutrophil Extracellular Trap Formation: A Matter of Mitochondrial Reactive Oxygen Species Generation and Citrullination and Cleavage of Histone. Front. Immunol..

[52] Santo-Domingo J., Demaurex N. (2010). Calcium Uptake Mechanisms of Mitochondria. Biochim. Biophys. Acta Bioenergy.

[53] Matsuyama S., Reed J.C. (2000). Mitochondria-Dependent Apoptosis and Cellular PH Regulation. Cell Death Differ..

[54] Zhao T., Wu W., Sui L., Huang Q., Nan Y., Liu J., Ai K. (2022). Reactive Oxygen Species-Based Nanomaterials for the Treatment of Myocardial Ischemia Reperfusion Injuries. Bioact. Mater..

[55] Mei H., Laws T.S., Mahalik J.P., Li J., Mah A.H., Terlier T., Bonnesen P., Uhrig D., Kumar R., Stein G.E. (2019). Entropy and Enthalpy Mediated Segregation of Bottlebrush Copolymers to Interfaces. Macromolecules.

[56] Teng C.-Y., Sheng Y.-J., Tsao H.-K. (2016). Boundary-Induced Segregation in Nanoscale Thin Films of Athermal Polymer Blends. Soft Matter.

[57] Wu D.T., Fredrickson G.H., Carton J.-P., Ajdari A., Leibler L. (1995). Distribution of Chain Ends at the Surface of a Polymer Melt: Compensation Effects and Surface Tension. J. Polym. Sci. Part B Polym. Phys..

