# Supplementary material for: Influence of Molecular Weight and Grafting Density of PEG on the Surface Properties of Polyurethanes and Their Effect on the Viability and Morphology of Fibroblasts and Osteoblasts
Source: Polymers (Basel). 2022 Nov 14;14(22):4912. doi: 10.3390/polym14224912 (PMC9698172; doi:10.3390/polym14224912)
Supplement: Supplementary file 1 [file polymers-14-04912-s001.zip › polymers-1952116-supplementary.pdf]

Supplementary Material

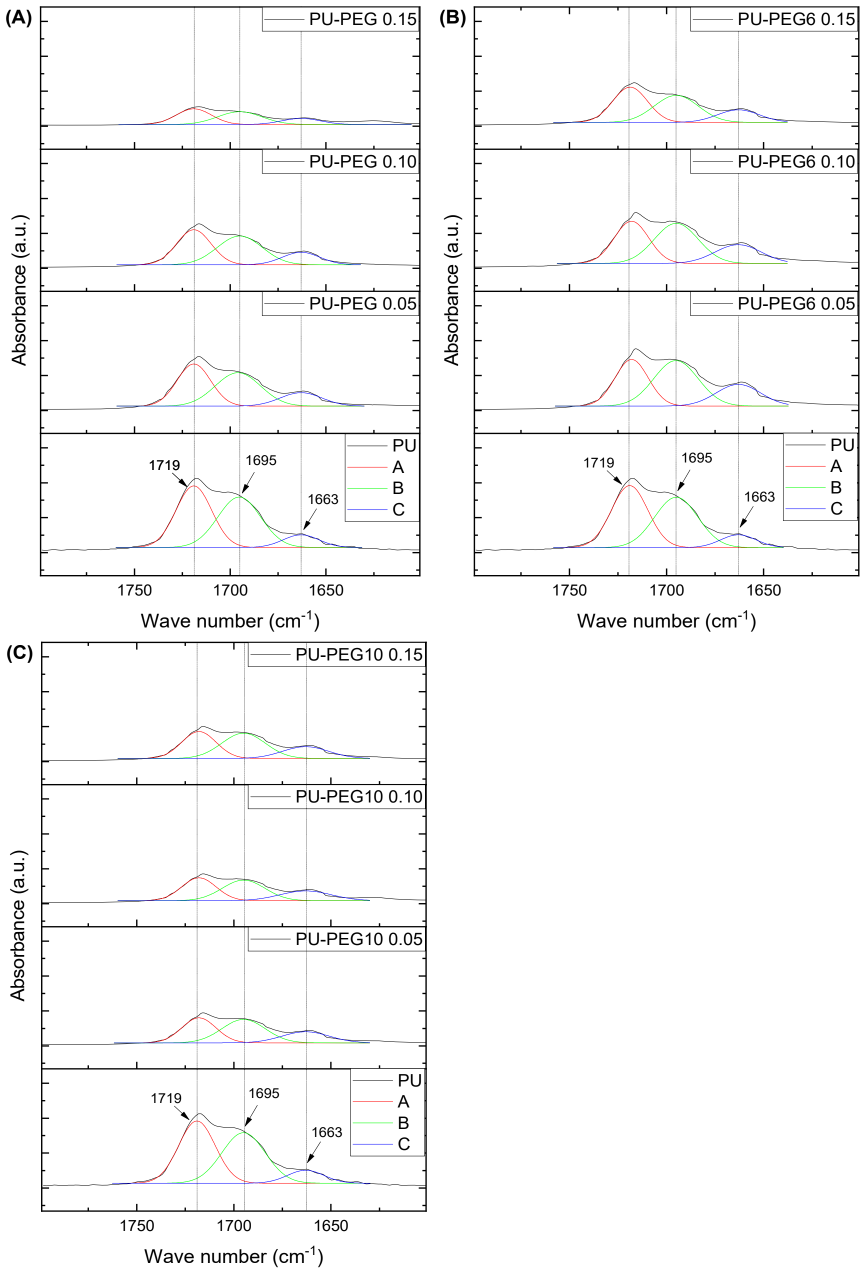

Figure S1. Deconvolution of the carbonyl bands from unmodified and PEG-grafted substrata. **(A)** PEG2-grafted PU; **(B)** PEG6-grafted PU; **(C)** PEG10-grafted PU.

Table S2. Band ratios of carbonyl bands from unmodified and PEG-grafted substrata.

Sample	C/(A+B)
PU	0.146
PU-PEG2 0.05	0.211
PU-PEG2 0.10	0.226
PU-PEG2 0.15	0.243
PU-PEG6 0.05	0.256
PU-PEG6 0.10	0.254
PU-PEG6 0.15	0.234
PU-PEG10 0.05	0.260
PU-PEG10 0.10	0.254
PU-PEG10 0.15	0.234

A: 1719 cm^{-1} band; **B:** 1695 cm^{-1} band; **C:** 1663 cm^{-1} band.

Table S3. Decomposition temperatures (Td) of PU, PEG and PEG-grafted substrata.

Sample	Td1 (°C)	Td2 (°C)
PU	340	413
PEG2	-	405
PU-PEG2 0.05	315	406
PU-PEG2 0.10	319	405
PU-PEG2 0.15	328	405
PEG6	-	413
PU-PEG6 0.05	330	407
PU-PEG6 0.10	319	406
PU-PEG6 0.15	334	415
PEG10	-	417
PU-PEG10 0.05	340	411
PU-PEG10 0.10	340	414
PU-PEG10 0.15	340	414

Table S4. pH measurements of culture medium (DMEM) with the PEG diluted.

	PEG amount (mg/mL)	PEG amount (μmol/mL)	pH
**DMEM**	0	0.0	8.50
**DMEM-PEG2**	1	0.5	8.57
	2	1.0	8.65
	3	1.5	8.70
**DMEM-PEG6**	1	0.2	8.57
	2	0.3	8.64
	3	0.5	8.70
**DMEM-PEG10**	1	0.1	8.57
	2	0.2	8.65
	3	0.3	8.70
